# Promyelocytic Leukemia with No Retinoic Acid Receptor Alpha Abnormality but with *RUNX1T1* Insertion to Chromosome 7q: A Classification and Management Dilemma

**DOI:** 10.1155/2015/412016

**Published:** 2015-08-17

**Authors:** Kathleen Overholt, Terri L. Guinipero, Nyla A. Heerema, Michael R. Loken, Samir B. Kahwash

**Affiliations:** ^1^Hematology/Oncology and Blood and Marrow Transplant, Nationwide Children's Hospital, Columbus, OH 43205, USA; ^2^Department of Pathology, The Ohio State University, Columbus, OH 43210, USA; ^3^Hematologics, Inc., Seattle, WA 98121, USA; ^4^Department of Pathology and Laboratory Medicine, Nationwide Children's Hospital, Columbus, OH 43205, USA

## Abstract

A case of acute promyelocytic leukemia (APL) with *RUNX1T1* insertion to 7q is described and compared to reported cases of APL with negative retinoic acid receptor alpha (*RARA*) abnormality. In this report, we describe the case of a 2-year-old boy who presented with bone pain and was found to have pancytopenia. Bone marrow examination showed morphologic and immunophenotypic findings typical of APL, but conventional cytogenetics, fluorescence in situ hybridization (FISH), and real-time polymerase chain reaction (RT-PCR) showed no evidence of *RARA* rearrangements. The only cytogenetic abnormality found was a small insertion in 7q, and three copies of *RUNX1T1*. Gene sequencing results became available after initiating therapy but were not informative. We describe the rarity of such cases and discuss how the typical morphologic and immunophenotypic findings of APL, coupled with the definite absence of *RARA* rearrangement (by FISH and RT-PCR), present a diagnostic and classification dilemma, raising the possibility of an unknown alternative mechanism for the leukemogenesis and maturation arrest seen in other APL variants. The diagnostic challenges and urgent management issues this unusual case raises may justify including it, along with similar cases, in a separate subtype of acute myeloid leukemia (AML) in future classifications.

## 1. Introduction

In this report, we describe a case of acute myeloid leukemia (AML) with morphologic and immunophenotypic findings typical of acute promyelocytic leukemia (APL), but conventional cytogenetics, fluorescence in situ hybridization (FISH), and real-time polymerase chain reaction (RT-PCR) showed no evidence of retinoic acid receptor alpha (*RARA*) abnormality. The only cytogenetic abnormality found was an additional copy of* RUNX1T1* inserted in 7q.

We discuss how the typical morphologic and immunophenotypic findings of APL, coupled with the definite absence of* RARA* rearrangement by FISH and RT-PCR, present a diagnostic and classification dilemma, raising the possibility of an alternative mechanism for the leukemogenesis and maturation arrest seen in APL. We also propose including cases of APL with no* RARA* abnormality in a separate subtype in future classifications of AML.

## 2. Case Report

A 2-year-old, previously healthy male presented with a one-month history of daily fevers (39°C or greater), fatigue, decreased oral intake, lower extremity pain, and intermittent refusal to bear weight. He was initially evaluated for possible left hip synovitis based on ultrasound findings. Laboratory findings at that time were significant for mild normocytic anemia (Hgb, 10.3 g/dL), elevated erythrocyte sedimentation rate (ESR) of 27, mildly elevated lactic dehydrogenase (LDH) of 1310 IU/L, and positive antinuclear antibodies (ANA) (1 : 40 in speckled pattern). One week later, he presented again with persistence of symptoms. Repeat lab tests showed similar results to the prior visit with persistent mild anemia (Hgb, 10.4 g/dL), normal general chemistry tests, normal coagulation tests, and elevated ESR of 58. Because of concern for a septic joint or neoplasia, the patient was admitted for evaluation.

During admission, MRI of pelvis and bilateral hips were performed. Results were significant for scattered areas of signal abnormality within the marrow of the pelvis and proximal left femur, with associated edematous or inflammatory changes within soft tissues of the pelvis. Findings were felt to be consistent with inflammatory or infectious etiology, although malignancy could not be excluded. The patient then underwent a bone marrow aspirate and biopsy; findings were felt to represent either aggressive marrow regeneration or early stages of a myeloproliferative process with no identified dysplastic features. Flow cytometric analysis demonstrated a predominant maturing myeloid cell population; however, only a very small population, ~2%, was phenotypic progenitor cells. The patient's symptoms were attributed to an inflammatory condition and the patient was discharged home on anti-inflammatory medication with primary care follow-up.

Symptoms initially improved, however, over the two weeks following discharge; his symptoms of fever and bone pain returned. He was seen in the Infectious Disease Clinic, where he was noted to have worsening anemia (Hgb of 7.2 g/dL) and thrombocytopenia (42,000 per microliter), with LDH elevation of 1661 and normal uric acid. He, therefore, underwent repeat bone marrow examination.

## 3. Pathologic Findings

At the time of this second bone marrow sample, the peripheral blood was significant for thrombocytopenia (platelet count of 35,000) with no anemia and normal white blood cell count; however, the patient had recently been transfused with packed red blood cells. The peripheral blood smear manual differential cell count listed 2% blasts. The bone marrow aspirate was hemodiluted with no significant marrow elements and no significant number of blasts.

**Figure 1:**
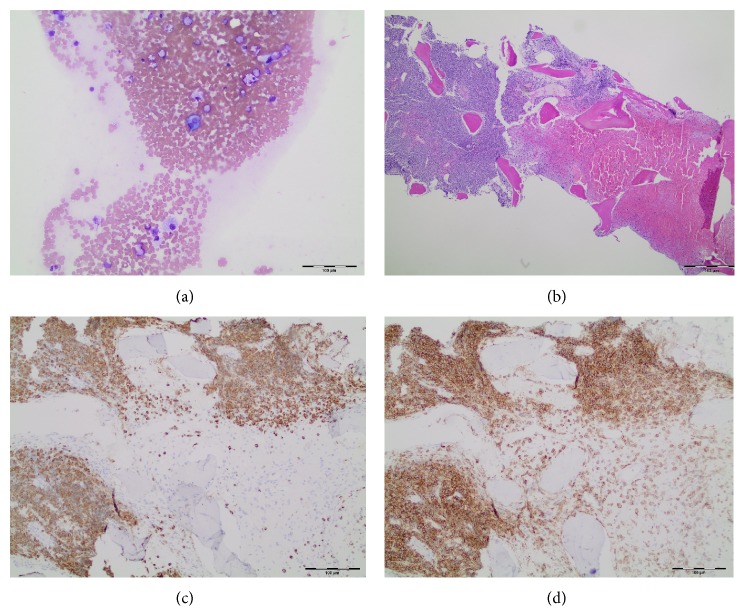
Initial core biopsy touch imprints. (a) Wright-Giemsa stained blood-dilute touch imprints; note the packed marrow on the left side. (b) Hemorrhagic necrosis on right side of this H&E stained section. (c) Immunoperoxidase staining of core biopsy for CD33; areas not taking the stain represent necrotic marrow. (d) Immunoperoxidase staining for Myeloperoxidase of a section from core biopsy.

**Figure 2:**
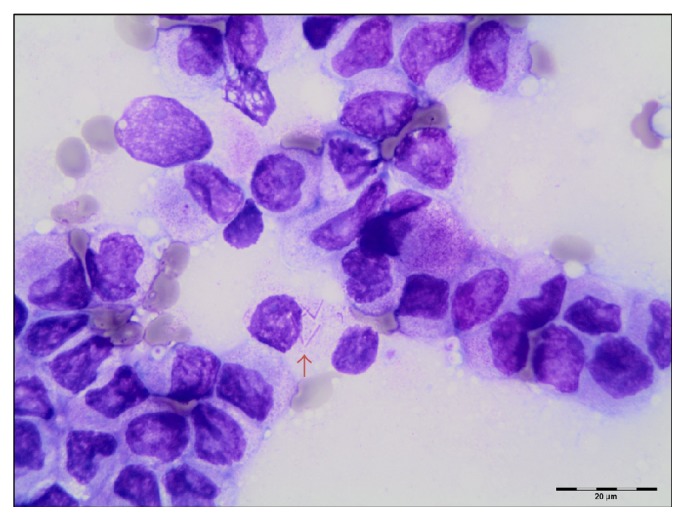
Wright-Giemsa stained touch imprint showing promyelocytes with fine azurophilic granules. Arrow: a myeloblast with multiple thin Auer rods typical of APL.

The core biopsy touch imprints also were paucicellular (Figure 1(a)), and flow cytometry showed no significant blast/progenitor cell population. Sections from the core biopsy were available the following day, revealing a hypercellular marrow space with an area of coagulative necrosis (Figure 1(b)). The marrow space was dominated by myeloid cells with some maturation and increased cells with cytoplasmic granules. There was a marked decrease in erythroid cells and megakaryocytes. Immunoperoxidase staining confirmed the predominance of myeloid cells (positive CD33 and MPO, Figures 1(c) and 1(d)). The negative staining for T-cell, B-cell, CD61, and CD42b helped exclude other hematopoietic malignancies, and negative results for mast cell tryptase, CD2, and CD25 excluded mastocytosis. A repeat marrow sampling including additional core biopsies was recommended in order to compensate for the inadequate blood-dilute aspirate and repeat immunophenotyping by flow cytometry on a more representative sample. Additional core biopsies were obtained, with one core used to generate a cell suspension of marrow cells by teasing out and disaggregating marrow tissue. This new cell suspension was used for making stained cytospin slides for cell morphology alongside the touch imprints and was utilized for immunophenotyping by flow cytometry and for cytogenetics. The touch imprints from this sample showed predominance of abnormal promyelocytes, with some showing multiple delicate Auer rods (arrow in Figure 2).

Immunophenotyping by flow cytometry revealed a predominance and abnormal expansion of phenotypic promyelocytes with no significant number of myeloid progenitor cells. The dominant abnormal cells were identified by high right angle scatter showing strong positivity for CD33 and Myeloperoxidase (MPO) but were negative for CD15, HLA-DR, and CD18 (abnormal findings). From this data, the diagnosis of APL was suspected. Molecular and cytogenetic studies were expedited, but no evidence of a* RARA* abnormality by karyotyping or FISH was seen. FISH with* PML-RARA* dual fusion and* RARA* break-apart probes (all probes from Abbott Molecular, Des Plaines, IL) were all normal. Metaphase cytogenetics showed a karyotype of 46,XY,ins(7;?)(q22;?). FISH with* D7Z1*,* D7S486*,* CBFB*, and* KMT2A* were also normal. However, although FISH with the* RUNX1T1-RUNX1* probes did not show typical fusion, three copies of* RUX1T1* were present. FISH on metaphases showed that* RUNX1T1* was present on the abnormal chromosome 7, resulting in a reinterpretation of the karyotype to 46,XY,der(7)ins(7;8) (q22;q13q22). A marrow specimen was sent for RT-PCR testing, which also did not show* RARA* abnormalities; however, testing was specific for only PML fusion partner. Given that morphologic and genetic findings were not quite clear, genetic sequencing was considered for further classification. However, by the time this testing was discussed, our patient had been started on treatment and initial bone marrow sample was no longer viable.

**Figure 3:**
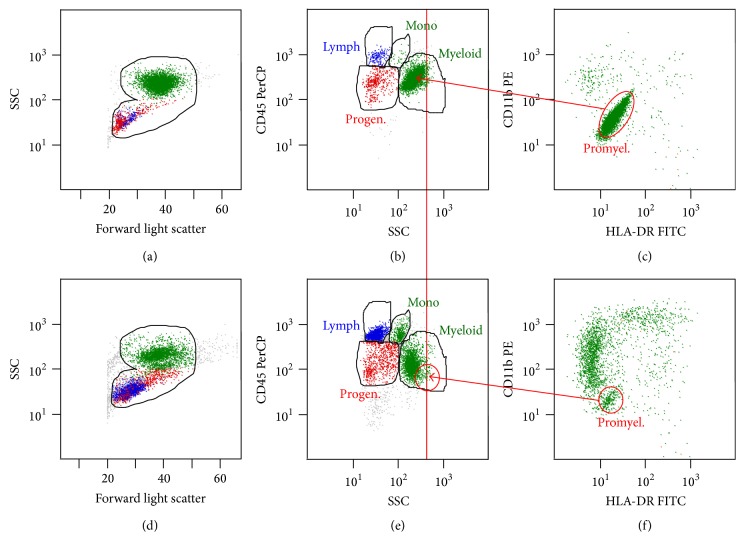
(a, b, c) Flow cytometric analysis of the bone marrow, following induction I chemotherapy. The dominant cell population, 56% of nonerythroid cells (green), was consistent with abnormal hypogranular promyelocytes that expressed neither CD11b nor HLA-DR but were highly autofluorescent. (d, e, f) Similar analysis of bone marrow, following induction II, demonstrated the appropriate right angle light scatter for promyelocytes (vertical red line) with the majority of maturing myeloid cells now expressing CD11b, that is, myelocytes-segmented neutrophils.

As the morphologic diagnosis of APL could not be confirmed by molecular and genetic methods, a treatment protocol for AML, not otherwise specified (NOS), was considered the best option. The patient was started on chemotherapy treatment per the standard arm of Children's Oncology Group AAML1031 protocol, although not enrolled in the study. Lumbar puncture was performed and no evidence of disease was present. Initial coagulation studies were within normal limits. However, after initiation of treatment, the patient developed bleeding symptoms consisting of epistaxis, guaiac positive stools, and oozing at the site of central line insertion despite maintaining a platelet count higher than 75,000. Repeat coagulation tests at that time revealed a mildly elevated prothrombin time (PT) of 15.2 (normal 12.4–14.7 seconds), a normal activated prothrombin time (aPTT) of 34 (normal 24–36 seconds), fibrinogen of 227 mg/dL (normal 170–410 mg/dL), elevated D-dimer of >20 (normal <0.50 mcg/mL), and a mildly elevated thrombin time of 33.6 (normal range 14.2–18.9 seconds). Due to a prior history of bleeding symptoms, a qualitative Factor XIII was performed and found to be within normal limits. von Willebrand panel was obtained on parents (since patient had received multiple blood products) and results returned within normal limits. The patient required multiple transfusions with platelets and fresh frozen plasma to control bleeding, resulting in symptoms improvement and count recovery. End of induction I bone marrow was performed due to slow count recovery and showed morphologic blasts at 15% and flow cytometry for measurable residual disease (MRD) identified an abnormal promyelocytes population at 56% (Figures 3(a)–3(c)) exhibiting decreased granularity and no expression of either HLA-DR or CD11b. The majority of phenotypic progenitor (blast region) cells were shown to be lymphoid (8.8% hematogones) and not myeloid. The myeloid progenitor cells were identified as normal phenotype with total of 2.7% CD34+ cells. At this time, given the persistence of an abnormal promyelocytic population, the sample was sent for DNA sequencing (Foundation One) for additional insight into this disease. Results showed one genomic alteration at CD36/Y325 of unknown significance.

After induction II per AAML1031 high risk arm bone marrow morphologic examination showed megaloblastoid maturation with 7% blasts; however, flow cytometric analysis revealed normal antigen expression on all myeloid precursors and absence of the abnormal promyelocytic population identified earlier. Flow cytometry revealed an MRD of less than 0.1% abnormal myeloid cells (Figures 3(d)–3(f)).

The patient started intensification I per AAML1031 high risk arm which he tolerated without issue. Due to his poor initial response, the bone marrow transplant team was consulted for stem cell transplant. At the time of publication of this paper, the patient has undergone allogenic stem cell transplantation with a 6 out of 6 matched cord donor and is doing well more than 100 days post-transplant.

## 4. Discussion

The current WHO classification of hematopoietic malignancies [1] does not include a defined subtype for APL cases lacking retinoic* RARA* abnormality or* RARA* variants. As a result, an encounter with one of the rare cases with definite morphologic and immunophenotypic features of APL but no* RARA* fusion gene (or its variants) may present a challenge of diagnosis, classification, and management. The rarity of such cases and the variable degrees of sensitivity in detecting the characteristic molecular abnormality add more complexity. Detailed documentation will help confirm the existence of such rare cases and further dispel the possibility that the absence of molecular findings is a result of insufficient work-up or technical failure.

FISH is the most widely used method for the detection of* RARA* rearrangements, as it is more sensitive than conventional karyotyping. Detection of* RARA* abnormalities by RT-PCR is usually the next and, in most instances, the last practical step for the few suspected cases of APL that fail FISH. There are fifteen reported cases of RT-PCR proven* RARA* rearrangements that failed FISH [2–6] and one reported case where* RARA* rearrangement was only found upon genetic sequencing [7].

Our case presented challenges at multiple levels starting with specimen adequacy and ending with the complexities of discordance between the morphologic and immunophenotypic findings on one hand and the molecular and genetics results on the other.

The initial bone marrow aspirate was suboptimal due to extreme blood dilution resulting from a combination of a “packed” marrow and partial marrow necrosis. This demonstrates the requirement for an adequate bone marrow specimen for proper diagnosis as AML cells may not circulate in the blood. The cell suspension obtained by disaggregating freshly obtained core biopsies was utilized to compensate for this inadequacy and was used to perform immunophenotypic analysis by flow cytometry and genetic studies.

A diagnostic and classification dilemma arose after the full morphologic, immunophenotypic, and genetic work-up was completed. While classifying this case as APL is appropriate based on the morphology and immunophenotype, this designation would open the possibility for insufficient management given that the lack of* RARA* abnormality correlates with no response to all trans retinoic acid (ATRA) therapy [8]. Alternatively, it is difficult to fit this case into another AML subtype based on morphology, as most of neoplastic cells are abnormal promyelocytes and not “real” myeloblasts. In fact, myeloblasts would not have reached the 20% threshold needed to diagnose most AML subtypes, if abnormal promyelocytes were not added to blast count.

**Figure 4:**
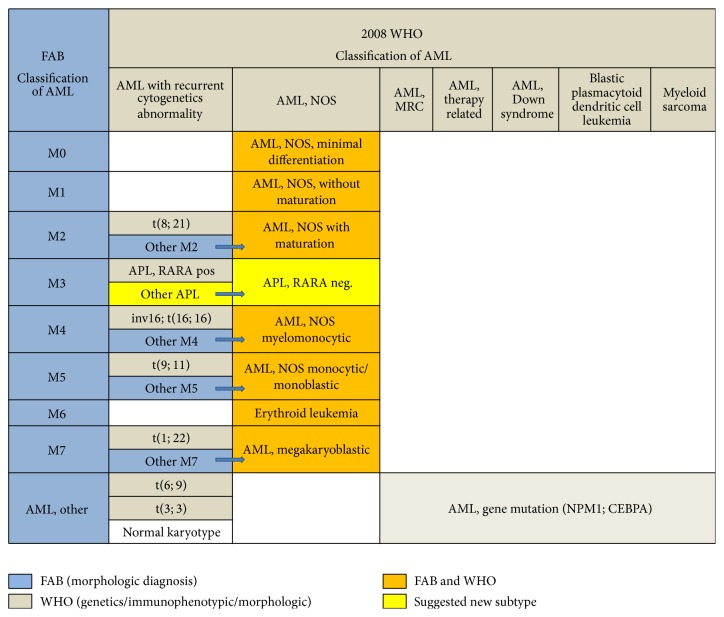
An approximate correlation between FAB and WHO 2008 classifications of AML subtypes, including suggested new subtypes “APL, NOS, and RARA negative.” FAB, French-American-British; WHO, World Health Organization; AML, acute myeloid leukemia; NOS, not otherwise specified; APL, acute promyelocytic leukemia; RARA, retinoic acid receptor alpha; MRC, myelodysplasia related changes.

Based on the facts illustrated by this case, it became evident that the current WHO classification has no appropriate subtype that would fit this case. Hence, there exists a need to create a subtype that accommodates such cases. This new category would help classify these rare cases in a manner analogous to classifying cases of Myelomonocytic Leukemia (AML-M4 in French-American-British classification or FAB) [9] as AML, NOS, Myelomonocytic (in World Health Organization classification or WHO), if they do not exhibit a recurrent cytogenetic abnormality. This is also similar to the manner cases of (AML-M2 in FAB) are classified as AML, NOS, myeloid with maturation (in WHO) after excluding t(8;21). An approximate correlation between the subtypes of the morphology-based FAB classification and the current WHO classification of AML, including the suggested subtype of APL with negative RARA, is shown in Figure 4. A suggested term for the new subtype is “APL, NOS, and* RARA* negative.”

Suggested criteria for inclusion in this new subtype are (1) morphologic and immunophenotypic features typical of APL, (2) myeloblasts and abnormal promyelocytes constituting a minimum of 20% of bone marrow cells, and (3) negative* RARA* by FISH and RT-PCR.

Clinically, the initial diagnostic dilemmas seen in this case resulted in treatment plan quandary, given that treatment and prognosis for APL and AML are very different. Given the compelling need to alleviate symptoms, it was felt that moving forward with induction per traditional AML therapy, while waiting for confirmatory testing, was the most appropriate course for this patient. While the patient did not have the classical clinical findings of* RARA* positive APL, he did also not fit the typical bone marrow morphologic findings of any other AML subtype. End of induction I bone marrow was performed due to slower than expected count recovery, and he was found to have positive MRD, as described earlier in this report. The new clinical onset of bleeding tendency and the pathologic findings were additional reasons to give pause and consider future treatment options. We proceeded per high risk standard AML treatment and moved forward with stem cell transplantation.

At the genetic level, the presence of three copies of* RUNX1T1* in this case supports a malignant process; however, the significance of this finding in the context of APL is not known. Whether the inserted gene is juxtaposed to another gene thus altering its expression is not known.

There are a few reported cases of morphologic APL that describe alternative translocations noted in the literature. Sainty et al. described work done by the European Working Group in 2000, where 90 cases of morphologic APL lacking the typical translocation t(15;17) were reviewed [10]. The results of the study indicated that, in the majority of cases (49 of 67 eligible cases), the traditional* PML-RARA* rearrangement could be identified by further molecular detection. The other 18 cases studied included 11 cases of morphologic APL with a t(11;17)(q23;q21) that coded for the* PLZF-RARA* fusion gene. These cases differed immunophenotypically from the traditional t(15;17) by the addition of CD56 positivity, and, clinically, these latter cases uniformly did not respond to ATRA therapy. One case was identified with t(5;17), resulting in the* NPM-RARA* rearrangement with no clinical or morphologic difference to typical t(15;17) APL and 1 case of unbalanced der(5)t(5;17). In 5 cases, no* RARA* rearrangements could be detected by RT-PCR, and these were under review at time of publication. These 5 cases were felt to represent mutations or alternations of pathways that mediate the differentiation of cells characteristic of APL either by mutation or epigenetic changes [10].

In addition to this large working study, there have been rare reports of other genetic alterations not including the* RARA* gene. A recent report by Duan et al. describes an adult patient with isolated i(17q) without t(15;17) and with negative* PML-RARA* by RT-PCR exhibiting morphology consistent with APL but responded poorly to traditional therapy [11].

Kim et al. reported one case of FISH negative APL with cryptic* PML-RARA* rearrangement detected by long distance PCR and noted only 12 such cases were reported in the literature at that time [2].

To our knowledge, there is no well documented case report of a typical APL that exhausted all levels of molecular detection of RARA and exhibited only an insertion of material from chromosome 8q in 7q as in this case. The diagnostic and management complexities that this and similar RARA negative cases present should justify creating a new AML subtype in future classification schema.
